# Review of fall risk assessment in geriatric populations using inertial sensors

**DOI:** 10.1186/1743-0003-10-91

**Published:** 2013-08-08

**Authors:** Jennifer Howcroft, Jonathan Kofman, Edward D Lemaire

**Affiliations:** 1 Department of Systems Design Engineering, University of Waterloo, 200 University Avenue West, Waterloo, ON N2L 3G1, Canada; 2 Ottawa Hospital Research Institute, Centre for Rehabilitation, Research and Development, 505 Smyth Road, Ottawa, ON K1H 8M2, Canada; 3 University of Ottawa, Faculty of Medicine, 451 Smyth Road, Ottawa, ON K1H 8M5, Canada

**Keywords:** Geriatric, Elderly, Older adults, Fall risk, Inertial sensor, Accelerometer, Gyroscope, Wearable sensor

## Abstract

**Background:**

Falls are a prevalent issue in the geriatric population and can result in damaging physical and psychological consequences. Fall risk assessment can provide information to enable appropriate interventions for those at risk of falling. Wearable inertial-sensor-based systems can provide quantitative measures indicative of fall risk in the geriatric population.

**Methods:**

Forty studies that used inertial sensors to evaluate geriatric fall risk were reviewed and pertinent methodological features were extracted; including, sensor placement, derived parameters used to assess fall risk, fall risk classification method, and fall risk classification model outcomes.

**Results:**

Inertial sensors were placed only on the lower back in the majority of papers (65%). One hundred and thirty distinct variables were assessed, which were categorized as position and angle (7.7%), angular velocity (11.5%), linear acceleration (20%), spatial (3.8%), temporal (23.1%), energy (3.8%), frequency (15.4%), and other (14.6%). Fallers were classified using retrospective fall history (30%), prospective fall occurrence (15%), and clinical assessment (32.5%), with 22.5% using a combination of retrospective fall occurrence and clinical assessments. Half of the studies derived models for fall risk prediction, which reached high levels of accuracy (62-100%), specificity (35-100%), and sensitivity (55-99%).

**Conclusions:**

Inertial sensors are promising sensors for fall risk assessment. Future studies should identify fallers using prospective techniques and focus on determining the most promising sensor sites, in conjunction with determination of optimally predictive variables. Further research should also attempt to link predictive variables to specific fall risk factors and investigate disease populations that are at high risk of falls.

## Introduction

Approximately one third of people over 65 years of age will fall each year [1,2], with the fall rate increasing with age [3,4] and for those in long-term care [5]. Elderly fall related injuries cost $20 billion per year in the United States alone [6]. Furthermore, the direct-care costs of fall related injuries could reach $32.4 billion per year by 2020 [7].

Falls can result in lasting and critical consequences; including, injury [3,8], long-term disability [9], reduced activity and mobility levels [4,8,10,11], admission to long-term care institutions [4,10,11], fear of falling [8,11], reduced self-confidence in mobility [4,12], and death [11,13]. Fear of falling is a particularly worrisome consequence since fear can lead to a cyclical pattern of mobility deterioration, social isolation, and decreased quality of life, even without a fall occurring [14].

The seriousness of the physical, psychological, and economic consequences of falling has led to two fall-management approaches. The first uses physical-monitoring devices to detect falls and signal for immediate care. However, this approach can only reduce consequence severity. The second approach prevents fall occurrence through interventions such as exercise [15,16], improved footwear [15], assistive devices [16], adaptation or modification of the home environment [15,16], review and modification of medication [15], and increased surveillance and care by caregivers [16]. Fall risk assessment is an important and effective prevention tool that identifies intrinsic (muscle weakness, neurological deficits, etc.) and extrinsic (poor lighting, inappropriate footwear, etc.) risk factors that help determine the most appropriate interventions, and ultimately reduce or eliminate falls [16].

Clinical fall risk assessments often involve questionnaires or functional assessments of posture, gait, cognition, and other fall risk factors [17]. These clinical assessments can be subjective, qualitative [17,18], and use threshold assessment scores to binarily categorize people as fallers and non-fallers. This oversimplifies geriatric fall risk, which is more accurately modeled by a continuum of fall risk with fuzzy boundaries between multiple risk categories, such as low, moderate, and high fall risk. Sensors that measure whole body motion [19], ground reaction forces [20], and electromyographic signals [21] provide objective, quantitative measures for fall risk assessment. However, the associated equipment is typically located in a gait laboratory and requires a time consuming setup that is difficult to practically integrate into typical clinic schedules. This limits the testing location and frequency. A wearable system that can efficiently capture and analyze quantitative mobility data could improve fall risk assessment.

Small wearable sensors can provide movement information during daily-living tasks, performed within real-world environments, instead of the simulated activities used in most clinical assessments. Gyroscopes and accelerometers are inertial sensors that are inexpensive, small, portable, and can be applied in common point-of-care environments or in the community (i.e., outdoors, stairs, ramps, etc.). Inertial sensors directly measure angular velocity and linear acceleration of body segments, from which other body motion parameters can be calculated. Inertial sensors in physical activity-monitoring systems have been used to detect falls, and several review papers summarize advances in this area [13,22-26].

Recently, inertial sensors have been incorporated into fall risk assessment protocols for older adults and could be used to generate input data for intelligent soft computing applications (i.e., methods that consider imprecision and uncertainty in complex system analysis) that represent geriatric fall risk as a continuum. To date, inertial sensor use in fall risk assessment has varied by study methodology, assessment variables, and fall risk models. One of the accelerometer-based physical-activity-monitoring review papers [26] provided only a brief review of inertial-sensor-based fall risk assessment. Shany et al. [27] provided an interesting discussion of wearable sensors for fall risk assessment, focusing on high-level methodologies when assessing structured or unstructured movements in supervised or unsupervised environments.

For inertial-sensor-based fall risk assessment, a critical and comprehensive examination is still required that addresses study methodologies, assessment activities, sensor locations on the body, measured and derived variables, functional clinical assessment comparators, and fall risk assessment model-validation methods. This paper performs such a critical examination of inertial sensor application for fall risk assessment and identifies important areas for future work.

### Search strategy and selection criteria

**Figure 1:**
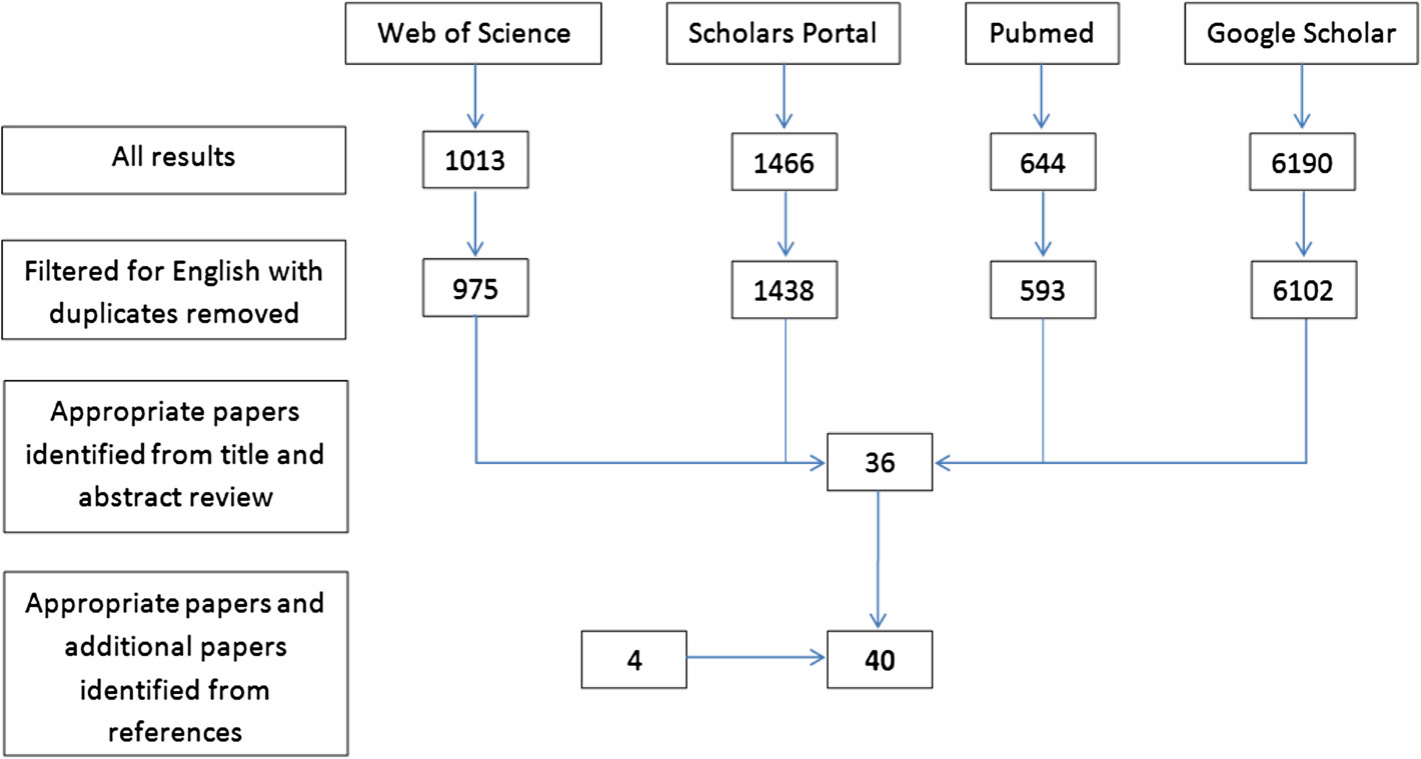
Summary of literature search process.

A literature review was performed using the key words: “(aged or geriatr* or gerontol* or senior* or elder* or old*) and (acceler* or inertia* or gyro*) and (fall* or fall risk or fall prediction)” on Web of Science, Scholars Portal, Pubmed, and Google Scholar on March 25 2013. Reference lists from the identified publications were reviewed to identify additional research articles of interest. The results of this search are shown in Figure 1. Paper inclusion criteria included fall risk assessment using inertial sensors, involvement of a geriatric population based on a mean participant age greater than or equal to 60 years, and published in English.

Forty articles from the database searches and article references met the inclusion criteria. Pertinent methodological features were extracted; including, fall risk classification method, sensor placement, activities assessed while inertial sensors were worn, and inertial-based parameters used to assess fall risk. Model assessment outcomes (i.e. accuracy, specificity, and sensitivity [28]) were extracted when available.

## Results

### Fall risk classification

Three main methods were used to classify subjects into faller and non-faller categories: retrospective fall history (30%), prospective fall occurrence (15%), and scores on clinical assessments (32.5%). A combination of retrospective fall history and clinical assessment tools were used to assess fall risk in 22.5% of the studies. Brief descriptions of the clinical assessment tools are provided in Table 1. Table 2 lists fall risk classification methods used in the literature.

**Table 1:** Clinical assessment tools

**Assessment tool**	**Description**
Barthel Index [29]	Ordinal scale that ranks subjects from 0 (total dependence) to 100 (total independence) based on 8 self-care and 2 mobility activities of daily living.
Fried’s Frailty Criteria [30]	Presence of 3 or more of 5 frailty indicators (significant and unintentional weight loss, grip weakness, poor endurance and energy, slow gait speed, low physical activity level).
Fukuda Test [31]	The person is blindfolded, extends both arms, and marches in place for 50 to 100 steps. Maximum body rotation greater than 30° indicates vestibular deficits.
Mini Motor Test [32]	20 item test that assesses abilities in bed (2 items), sitting position (3 items), standing position (9 items), and gait (6 items).
One Legged Stance Test [33]	Time a person can stand on one leg without upper extremity support and without bracing the suspended leg against the stance leg. Greater than 30 s indicates low fall risk and less than 5 s indicates high fall risk.
Physical Performance Test [34]	Ability to stand with feet together side-by-side, semi-tandem, and tandem; walk 8 ft; and rise from a chair and return to seated position.
Physiological Profile Assessment (PPA) [35]	Assessment of vision, peripheral sensation, muscle force, reaction time, and postural sway. Score of 0-1 = mild risk, 1-2 = moderate risk, and >2 = high risk of falling.
STRATIFY Score [36]	Assessment of 2 month fall history, mental alteration, frequent toileting, visual impairment, psychotropic medication use, and mobility issues. Score of <2 indicates increased fall risk.
Timed Up and Go (TUG) [37]	Time to stand up from an armchair, walk 3 m, turn, walk back to the chair, and sit down again. Times that exceed 14 s indicate increased fall risk for community dwelling elderly without neurological disorders.
Tinetti Assessment Tool [38]	Dynamic balance and gait evaluation with 10 balance components and 8 gait components. Overall scores <19 = high fall risk, 19-23 = moderate fall risk, > 23 = low fall risk. Maximum score = 40.

**Table 2:** **Criterion classification methods used to assess inertial**-**sensor**-**based fall risk measures**

	**Retrospective history**	**Prospective occurrence**	**Assessment tools**
Auvinet et al., 2003 [39]	1 year	-	-
Bautmans et al., 2011 [40]	6 months	-	TUG >15 s or Tinetti score ≤24
Caby et al., 2011 [41]	1 year	-	25 m walking, Mini Motor test, Tinetti test, TUG, Physical Performance Scale, Fukuda test, One Legged Stance test
Cho and Kamen 1998 [18]	1 year	-	Self-reported frequent fallers
Doheny et al., 2011 [42]	5 years	-	Self-reported fear of falling or presence of cardiovascular risk factors
Doheny et al., 2012 [43]	5 years	-	-
Doi et al., 2013 [44]	-	1 year (reported weekly)	-
Ganea et al., 2011 [45]	-	-	Fried’s criteria for frailty
Giansanti et al., 2006, 2008 [46-48]	Unspecified	-	Tinetti test level 3
Gietzelt et al., 2009 [49]	-	-	STRATIFY score (includes 2 month fall history) ≥2
Greene et al., 2010, 2012 [50,51]	5 years	-	-
Ishigaki et al., 2011 [52]	-	-	One Legged Stance test (eyes open) ≤15 s and/or TUG ≥11 s
Kojima et al., 2008 [53]	1 year	-	-
Laessoe et al., 2007 [54]	-	1 year (fall diary with contact at 6 months)	-
Latt et al., 2009 [55]	1 year	-	-
Liu et al., 2008 [56]	Unspecified	-	Falling during gait perturbation assessment, medical history, self-identification as frequent faller
Liu et al., 2011 [57]	-	-	PPA
Liu et al., 2011 [58]	1 year	-	-
Marschollek et al., 2008 [59]	-	-	TUG > 20 s, STRATIFY score >2, Barthel Index: Mobility score <10
Marschollek et al., 2009 [60]	In-hospital history	-	-
Marschollek et al., 2011 [61,62]	-	1 year	-
Martinez-Ramirez et al., 2011 [63]	-	-	Body mass loss ≥4.5 kg, low energy, low physical activity, weakness, slowness
Menz et al., 2003 [64]	-	-	Overall fall risk score (low, moderate, high risk) based on vision, peripheral sensation, strength, reaction time, balance tests
Moe-Nilssen et al., 2005 [65]	1 year	-	-
Najafi et al., 2002 [17]	-	-	Fall risk score ≥5 based on balance, gait, visual, cognitive and depressive disorders, history of falls.
Narayanan et al., 2008, 2009, 2010 [66-68]	-	-	PPA
O’Sullivan et al., 2009 [1]	1 year	-	-
Paterson et al., 2011 [69]	-	1 year (reported monthly)	-
Redmond et al., 2010 [70]	-	-	PPA
Schwesig et al., 2012 [71]	-	1 year (recorded by caregivers)	-
Senden et al., 2012 [72]	-	-	Tinetti test ≤24 (Low risk 19-24, High risk <19)
Toebes et al., 2012 [73]	1 year	-	-
Weiss et al., 2011 [74]	1 year	-	-
Yack and Berger [75]	1 year	-	Self report of unsteady or unstable walking and/or standing

Assessment tool thresholds indicate a high fall risk category.

### Inertial sensors

Two different inertial sensors were used in the reviewed papers: gyroscopes (angular velocity) and accelerometers (linear acceleration) [76]. Descriptions of gyroscopes and accelerometers are provided by Webster [76]. Accelerometers were the only inertial sensor in 70% of the studies, whereas gyroscopes were the only inertial sensor in one study [17]. Both accelerometers and gyroscopes were used in 27.5% of the studies.

### Sensor location

Accelerometers and gyroscopes are small enough for measurement during activity, via attachment to a body part, belt, or headband. The lower back, including the pelvis, sacrum, and the L3 to L5 vertebrae, is the most common sensor location and was the only location in 65% of the studies. This site approximates the center of mass location [46,49,60,61,74] and is acceptable for long-term at-home use [49,61]. Other sensor locations include the head [18,55,64], upper back [44,73,75], sternum [17,42,45], shoulder [41], elbow [41], wrist [41], hip [18,56], thigh [42], knee [41,56], shank [50], ankle [41,56], and foot [69,71].

### Assessed activity

Various activities were used for inertial-sensor-based fall risk assessment. The most frequently assessed activity was level ground walking (45%), followed by Timed Up and Go (TUG) (32.5%), sit-to-stand transitions (STS, 22.5%), standing postural sway (20%), left-right Alternating Step Test (AST) on level ground (15%), and uneven-ground walking (2.5%). Many studies used a combination of activities (20%). For level walking, subject-selected walking speed was assessed in the majority of studies (66.7%) while other walking speeds (slow, fast) were assessed in 26.7% of studies and a designated speed was assessed in one study [73].

### Variables

One hundred and thirty distinct variables were assessed in the literature, and can be categorized as: position and angle (7.7%), angular velocity (11.5%), linear acceleration (20%), spatial (3.8%), temporal (23.1%), energy (3.8%), frequency (15.4%), and other (14.6%). All variables that had significant outcomes (p < 0.05), together with sensor body locations, are presented in Table 3.

**Table 3:** Significant inertial-sensor-based variables (p < 0.05) with associated sensor location

**Category**	**Variable**	**Sensor location**
**Position and Angle Variables**	AP peak to peak amplitude	LB [52]
	ML peak to peak amplitude	LB [52]
	V peak to peak amplitude	LB [52]
	AP and ML postural sway length during stance	LB [43,51]
	Trunk tilt	St [44]
**Angular Velocity Variables**	Min, mean, max AP	Sha [50]
	Min, mean, max ML	Sha [50]
	Min, mean, max V	Sha [50]
	AP peak to peak amplitude	LB [52]
	ML peak to peak amplitude	Sha [50]
	V peak to peak amplitude	LB [52]
	Postural sway velocity during stance	LB [43,51]
	Mean squared modulus ratio for postural sway	LB [46-48]
	AP RMS during stance	LB [51]
	ML RMS during stance	LB [51]
	V RMS during stance	LB [51]
	3D RMS during stance	LB [51]
	ML variability	UB [73]
**Linear Acceleration Variables**	Median AP	LB [74]
	SD of AP	He [18], LB [74], Hi [18]
	Peak AP	UB [75]
	Peak V	UB [75]
	AP peak to peak amplitude	LB [52]
	ML peak to peak amplitude	LB [49,52,60]
	V peak to peak amplitude	LB [52]
	AP RMS	He [55], LB [55]
	ML RMS	He [55], LB [55]
	V RMS	He [55], LB [55,71]
	AP RMS during stance	LB [51]
	ML RMS during stance	LB [51]
	V RMS during stance	LB [51]
	2D RMS (ML and AP) during stance	LB [43]
	3D RMS	LB [1,57]
	3D RMS during stance	LB [51]
	Jerk	St [42]
	Sit to stand AP range	LB [74]
	Stand to sit AP range	LB [74]
	Sit to stand Jerk	LB [74]
	Dissimilarity of AST subcomponents	LB [57,67]
	Dissimilarity of STS subcomponents	LB [67,68,70]
**Spatial Variables**	Number of steps	LB [60], Sha [50]
	Step length	He [64], LB [60,64,72]
**Temporal Variables**	Gait Speed	He [55,64], LB [40,44,55,64,72,74]
	Cadence	He [64], LB [39,58,64], Kn [41], Sha [50], An [41]
	Step duration	LB [74], Sha [50]
	Step duration variability	He [55], LB [55]
	Stride time	Fo [71]
	SD of stride time	Fo [71]
	% GC double support	Sha [50]
	TUG time	LB [66,74], Sha [50]
	TUG subcomponent time	LB [57,66], Sha [50]
	TUG: number of gait cycles	Sha [50]
	STS time	LB [66]
	STS subcomponent times	LB [57], Th [42]
	SD of STS subcomponent times	LB [66]
	Normalized SD of STS subcomponent times	LB [58,66]
	Sit/stand transition duration	St [17,45]
	Sit/stand SD of transition duration	St [17]
	AST time	LB [66]
	AST subcomponent times	LB [57,58,68,70]
	SD of AST subcomponent times	LB [66]
	Normalized SD of AST subcomponent times	LB [67,68,70]
**Energy Variables**	Kinetic Energy	LB [49]
	Local wavelet energy	St [45]
	Summed magnitude area of acceleration	LB [57,58,67]
	25% quartile frequency	He [18], Hi [18]
	50% quartile frequency	He [18], Hi [18]
	75% quartile frequency	He [18], Hi [18]
	Sway frequency during stance	LB [51]
	Number of FFT peaks	LB [61,62]
	Dominant FFT peak parameters	LB [59,61,62]
	1^st^ FFT peak parameters	Sho [41], El [41], Wr [41], Kn [41], An [41]
	Ratio of magnitude of even harmonics to odd harmonics	He [55,64], UB [44,75], LB [44,50,52,53,59,72]
	Area under 1^st^ 6 harmonics divided by remaining area	LB [57,58]
	Ratio of 1^st^ 4 harmonics to magnitude of 1^st^ 6 harmonics	LB [57,58]
	ML spectral edge frequency	St [42]
	Entropy of power spectrum	LB [53]
	Correlation between left and right arm signals	Sho [41], El [41], Wr [41]
	Maximum V acceleration Lyapunov Exponent	Hi [56], Kn [56], An [56]
	Maximum AV Lyapunov Exponent	UB [73], Hi [56], Kn [56], An [56]
	Autocorrelation coefficients of acceleration signal	LB [39,65]
	Trunk level forces	St [45]
	Continuous wavelet transform	LB [63]
	Discrete wavelet transform	St [45], LB [63]
	Detrended fluctuation fractal scaling index of acceleration derived stride time	Fo [69]
	Fractal dimension of acceleration versus AV	St [45]
	Number of abnormal sit/stand transitions	St [17]

*An*, ankle, *AP*, anteroposterior, *AST*, Alternating Step Test, *AV*, angular velocity, *COP*, center of pressure, *CoV*, coefficient of variation, *El*, elbow, *FFT*, Fast Fourier Transform, *Fo*, foot, *He*, head, *Hi*, hip, *GC*, gait cycle, *Kn*, knee, *LB*, lower back, *ML*, mediolateral, *RMS*, Root Mean Square, *SD*, standard deviation, *Sha*, shank, *Sho*, shoulder, *St*, sternum, *STS*, sit-to-stand transitions, *Th*, thigh, *TUG*, Timed Up and Go, *UB*, upper back, *V*, vertical, *Wr*, wrist.

Of the variables that were assessed in more than one study, only 13 were significant (p < 0.05) each time they were assessed: 1) mediolateral and anteroposterior postural sway length [43,51]; 2) mediolateral and anteroposterior postural sway velocity [43,51]; 3) ratio of mean squared modulus for postural sway [46-48]; 4) standard deviation of anteroposterior acceleration [18,74]; 5) root mean square amplitude of vertical linear acceleration [55,72]; 6) gait speed [40,44,55,64,72,74]; 7) sit-to-stand transition duration [17,45]; 8) dominant Fast Fourier Transform (FFT) peak parameters derived from lower-back linear acceleration signals [59,61,62]; 9) ratio of even to odd harmonic magnitudes derived from head, upper back, and lower-back linear acceleration signals [44,55,57,58,64,72,75]; 10) area under the first six harmonics divided by the remaining area for lower-back linear acceleration signals [57,58]; 11) ratio of the first four harmonics to the magnitude of the first six harmonics for lower-back linear acceleration signals [57,58]; 12) maximum Lyapunov exponent of angular velocity signal [73,77]; 13) discrete wavelet transform parameters from lower-back angular velocity and linear acceleration signals and sternum linear acceleration signals [45,63]. Six of these multi-study variables (1,2,5,6,9,12) were from different research groups, while seven variables (3,4,7,8,10, 11,13) were from a single research group.

Mediolateral and anteroposterior postural sway length and velocity are measures of postural stability that represent trunk movement during static standing [43,51]. The root mean square of vertical linear accelerations has been used to measure gait smoothness, with larger values linked to increased fall risk [55,72]. Low gait speed has been identified in the broader literature as an indicator of fall risk [77,78]; however, adoption of a low gait speed may also be an accommodation linked to fear of falling and not an indicator of high risk of falling [79]. The ratio of even to odd harmonics of linear acceleration reflects the proportion of the acceleration signal that is in phase with the participant’s stride frequency, with even harmonics correlating with in-phase components and odd harmonics correlating with out-of-phase components [55,75]. The area under the first six harmonics divided by the remaining area under the magnitude spectrum curve of the lower-back linear accelerations provides a quantification of gait periodicity [57,58], and the ratio of the first four harmonics to the magnitude of the first six harmonics for lower-back linear accelerations indicates the pattern of harmonic magnitudes [58]. The maximum Lyapunov exponent of angular velocity reflects local dynamic stability [56,73] and converts time series measurements into a state space [73]. In the state space, the Lyapunov exponents measure the average rate of expansion or contraction of the original trajectory in response to perturbations. Larger Lyapunov exponent values indicate a decreased ability to compensate for local perturbations during gait, increased instability, and increased fall risk [56,73].

A wide range of variables have been incorporated into fall risk classification models. Variables that achieved high fall risk classification sensitivity and specificity (i.e., greater than or equal to 80%) are discussed here. Successful single-variable models used: 14) the mean squared modulus ratio for postural sway derived from lower back angular velocity [46-48], 15) sit-to-stand transition duration determined from sternum acceleration and angular velocity [45], 16) sit-to-stand fractal dimension derived from sternum acceleration and angular velocity [45], and 17) sit-to-stand lower back jerk (derivative of acceleration) [74]. The mean squared modulus ratio for postural sway is the ratio of the rotational kinematic energy during eyes-open or closed postural sway on a foam surface, to the rotational kinematic energy during eyes-open postural sway on a firm surface [46-48]. The sit-to-stand fractal dimension represents the regularity of the sit-to-stand movement, with larger values associated with greater movement irregularity and fall risk [45].

Several multi-variable models also achieved sensitivity and specificity levels greater than or equal to 80%. One model included 18) pelvic sway and 19) kinetic energy [49]. Caby et al., 2011 [41] investigated a variety of single-variable and multi-variable models derived from accelerometers on the knee, ankle, shoulder, elbow, and wrist. These variables included correlation between left and right elbow accelerations, step frequency, jerk, spectral entropy, acceleration frequency parameters, and 25 m walk time normalized to participant height. Liu et al., 2011 [57] developed a multi-variable model that included 126 temporal, energy, and frequency variables derived from TUG, STS, and AST movements.

### Classification models of fall risk prediction

In half of the papers, geriatric fall risk was predicted using derived models (50%), as opposed to correlating a variable with fall risk or fall occurrence. Regression models (65%), mathematical classifiers (25%), decision trees (15%), neural networks (15%), support vector machines (10%), and cluster analysis (10%) were employed to predict fall risk, with some studies using more than one method (30%). The accuracy, specificity, and sensitivity [28] of these models are shown in Table 4.

**Table 4:** **Fall**-**risk assessment model type**, **validation method**, **accuracy**, **specificity**, **and sensitivity**

**Author**	**Model**	**Model validation**	**Accuracy (%)**	**Specificity (%)**	**Sensitivity (%)**
Caby et al., 2011* [41]	Radial basis function neural network, support vector, *k*-nearest neighbour, and naive Bayesian classifiers	Leave-one-out cross-validation	75-100	40-100	93-100
Giansanti et al., 2008*† [48]	Multi-layer perceptron neural network	47:53 split (Train:Test)	97	97	98
Giansanti et al., 2006*† [46]	Mahalanobis cluster analysis	47:53 split (Train:Test)	93.5-94.5	93-94	93.9-94.9
Giansanti et al., 2008*† [47]	Multi-layer perceptron neural network	47:53 split (Train:Test)	88-91	88-92	88-91
Gietzelt et al., 2009* [49]	Decision tree	Not specified	90.5	91.0	89.4
Ganea et al., 2011* [45]	Logistic regression, ROC curve	Not specified	-	35-88	55-92
Weiss et al., 2011† [74]	Logistic regression	Not specified	63.4-87.8	50.0-83.3	65.2-91.3
Liu et al., 2011* [57]	Linear regression, linear discriminant classifier	Leave-one-out cross-validation	71	98.3	88.9
Marschollek et al., 2011‡ [61]	Logistic regression, decision tree	Stratified ten-times ten-fold cross validation	78-80	82-96	58-74
Marschollek et al., 2008* [59]	Logistic regression, classifier	Stratified ten-times ten-fold cross validation	65.5-89.1	15.4-60.4	78.5-99.0
Marschollek et al., 2009† [60]	Decision tree	Not possible due to limited sample size	90	100	57.7
Schwesig et al., 2012‡ [71]	Binary logistic regression, ROC curve	Not specified	-	42-61	63-100
Moe-Nilssen et al., 2005† [65]	Linear regression, ROC curve	Not specified	80	85	75
Bautmans et al., 2011† [40]	Logistic regression, ROC curve	Not specified	77	78	78
Greene et al., 2010† [50]	Logistic regression	80:20 split (Train:Test)	76.8	75.9	77.3
Doi et al., 2013‡ [44]	Logistic regression, ROC curve	Not specified	-	84.2	68.8
Marschollek et al., 2011‡ [62]	Logistic regression, classifier	Stratified ten-times ten-fold cross validation	70	78	58
Greene et al., 2012† [51]	Support vector machine	Ten-fold cross validation	71.5	68.4	65.4
Kojima et al., 2008† [53]	Regression, canonical discriminant classifier	Not specified	62.1	68.2	61.1
Senden et al., 2012* [72]	Linear regression, ROC curve	Not specified	AUC: 0.67-0.85	-	-

*AUC*, Area under curve, *ROC*, receiver operating characteristic, Criterion classification method: *Clinical assessment, †Retrospective fall history, ‡Prospective fall history.

## Discussion

Wearable inertial sensors are a viable technology for fall risk assessment, joining clinical and laboratory methods as acceptable assessment tools. Inertial-sensor-based systems have the benefits of portability, low cost, and few constraints on the types of movements that can be monitored. These benefits are encouraging for real-world fall risk assessment applications.

### Fall risk assessment models

A wide range of sensitivity (55-100%) and specificity (15-100%) levels have been reported for inertial-sensor-based fall risk assessment models (Table 4). The highest sensitivity and specificity levels for retrospective fall history were 91.3% and 83.3%, respectively, and the highest levels using prospective fall history were 74% and 82%, respectively. However, 50% of the studies did not employ separate data sets for model training and validation, thereby limiting the model’s applicability beyond the training set population. Validating the model with the training population would likely result in inflated accuracy, specificity, and sensitivity levels when the model is applied to the general population compared to model validation with a different testing population. The five best performing models, in terms of overall accuracy, specificity, and sensitivity, used neural networks [47,48], naive Bayesian classifier [41], Mahalanobis cluster analysis [46], and a decision tree [49]. The five worst performing models used regression [44,53,62,72] and a support vector machine [51]. Therefore, intelligent computing methods (neural networks, Bayesian classifiers, etc.) may be more appropriate for fall risk classification than regression techniques.

In the majority of papers, sensors were placed on the lower back. However, the justification for this location is limited to an intention to approximate the body center of mass and the location’s unobtrusive nature for long-term use. High subject acceptance for long-term lower back sensor placement was found in a 20 day case-study by Giansanti et al., 2009 [80]. While the lower back is a promising sensor location, the upper back, hip, and thigh have potential as long-term sensor locations [81]. To date, there has been no objective evaluation to determine which sensor sites, or combinations of sites, provide the most reliable fall risk assessment. Other sensor locations, along the legs and arms, were used in predictive models with comparable sensitivity and specificity [41,50]. Current research has not confirmed if the total body center of gravity region is superior to other locations for fall risk identification.

Of the 13 variables that were assessed in more than one study and had significant outcomes in each study (p < 0.05) and the six variables that had high fall risk classification sensitivity and specificity, 58% were related to postural instability and gait consistency over strides (1–3,9-14,16,18). Further research is needed to corroborate these initial findings toward identifying a set of inertial-sensor-based variables that yield a robust and accurate fall risk assessment model and clinical tool.

### Criterion classification methods

Clinical fall risk assessment was the predominate criterion method for classifying fallers and non-fallers when evaluating sensor-derived fall risk classification, with 32.5% of the studies using clinical assessment as the only means of classification. However, clinical assessments include false positives and false negatives that introduce inaccuracies when evaluating sensor-based systems. Another concerning aspect is that fall risk thresholds were not used consistently across research studies. For example, three studies that assessed geriatric populations devoid of neurological disorders used different TUG completion-time thresholds to determine fall risk, ranging from 11 s to 20 s [40,52,59]. The literature recommends a threshold of 14 s for studies focused on community dwelling elderly without neurological disorders [37] and 30 s for community dwelling elderly with neurological disorders [82].

Retrospective fall assessment was the only criterion classification method in 30% of the studies; however, a fall would already have occurred before the study assessment. The gait strategies and patterns during the fall may have been different from those during the assessment, because the participants could have adjusted their walking and mobility patterns to be more conservative, stable, and safe. Prospective fall occurrence is the preferred criterion classification method when placing subjects into low risk (non-faller) and high risk (faller) categories, but prospective risk was only used in 15% of studies. Since the goal of fall risk assessment is to predict the likelihood of future falls, a prospective study that uses fall occurrence records after assessment would be more appropriate. Furthermore, prospective fall assessments have greater accuracy compared to retrospective fall assessment due to patient fall recollection issues with retrospective assessment [83].

### Outlook for future work

A thorough assessment of optimal inertial sensor sites should be performed in conjunction with identification of optimal inertial-sensor-based variables, since the usefulness of a variable will likely be site specific. Better knowledge of user compliance with sensor placement at different sites will also be important when developing assessment tools that are widely accepted in the clinical and community environments. A generalized focus-group assessment of user acceptance of wearable sensors for fall detection determined that wearable sensors would be accepted provided they were unobtrusive [84]. Only one case study demonstrated the acceptability of the lower back sensor location [80]. Therefore, larger, long-term, user compliance studies of wearable sensors are needed to ensure feasibility for fall risk assessment. If long-term compliance is an issue, sensor use may be confined to short periods in a clinical environment.

Future studies should prospectively assess fall risk instead of relying on clinical assessments or a retrospective fall assessment, as previously discussed. Several papers emphasized the importance of using prospective fall risk occurrence in their future research [51,54,60,62,72-74], thereby avoiding the two biggest limitations of retrospective fall assessment: inaccurate recollection of fall history and gait changes due to past falls.

An important next step in this research field is to expand assessments from the geriatric population to include more specialized populations; including, those with Alzheimer’s and Parkinson’s diseases. Inertial sensors have the potential to identify patients with these diseases in the early stages, as suggested by Giansanti et al., 2008 [47], and identify those at risk of falling. One paper identified fallers from non-fallers in a Parkinson’s Disease elderly subgroup [55]. However, more disease-specific research is needed since optimal sensor site(s) and sensor features may be different from the generalized geriatric population. For example, arm swing may be a critical feature for people with Parkinson’s Disease because reduced arm swing is one of the first presenting gait impairment features [85].

Another critical area for future work is to match predictive variables with specific fall risk factors [58]. These fall risk factors could include physical factors such as peripheral neuropathy, muscle weakness, visual impairments, reduced flexibility, and lower limb arthritis. This is a crucial step that would increase the clinical value of inertial-sensor-based fall risk assessment tools and allow identification of specific impairments that increase fall risk, thus enabling individualized treatment and intervention. The walking environment (i.e. level ground, stairs, ramps) and the activity performed (i.e. transition from sitting to standing, walking outdoors) could be associated with increased fall risk. Identification of such high-risk environments and activities may increase the accuracy of fall risk assessment [86]. Identification of specific risk factors is clinically important for preventing falls and their sequelae [79].

To demonstrate the utility of inertial-sensor-based fall risk assessments, a comparison between inertial-sensor-based assessments and current clinical assessments must be made in relation to prospective fall occurrences. This would determine whether inertial-sensor-based fall risk assessment methods alone can provide better accuracy than current clinical assessments, whether a combination of inertial sensors and current clinical assessment would optimize fall risk prediction, and if specific risk factors can be better identified when using inertial-sensor-based information. While some work has been done comparing inertial-based assessment to clinical assessments [1,50], and using a combination of inertial and clinical assessments [60,61], more research in this area is required to conclusively demonstrate the advantages of inertial-based fall risk assessments.

## Conclusions

Inertial sensors have the potential to provide a quantitative, objective, and reliable indication of fall risk in the geriatric population, as demonstrated in the reviewed studies. High levels of accuracy, specificity, and sensitivity have been achieved in fall risk prediction models. Future studies should identify fallers using prospective techniques and focus on determining the most promising sensor site(s), in conjunction with determination of optimally predictive variables. Further research should also investigate disease populations that are at high risk of falls and link predictive variables to specific fall risk factors, including disease-specific factors.

## Abbreviations

An: Ankle; AP: Anteroposterior; AST: Alternating step test; AV: Angular velocity; COP: Center of pressure; CoV: Coefficient of variation; El: Elbow; FFT: Fast Fourier Transform; Fo: Foot; He: Head; Hi: Hip; GC: Gait cycle; Kn: Knee; LB: Lower back; ML: Mediolateral; PPA: Physiological profile assessment; ROC: Receiver operating characteristic; SD: Standard deviation; Sha: Shank; Sho: Shoulder; St: Sternum; STS: Sit-to-stand transitions; Th: Thigh; TUG: Timed Up and Go; UB: Upper back; V: Vertical; Wr: Wrist.

## Competing interests

The authors declare that they have no competing interests.

## Authors’ contributions

JH reviewed the literature and drafted the manuscript with JK and EL providing input on the manuscript content details including technical and clinical aspects. JH, JK, and EL revised and edited the manuscript. JH, JK, and EL read and approved the final manuscript.
